# Supplementary material for: Osmotic conditions shape fitness gains and resistance mechanisms during E. coli and T4 phage co-evolution
Source: mBio. 2026 Aug 17;17(9):e01167-25. doi: 10.1128/mbio.01167-25 (PMC13559767; doi:10.1128/mbio.01167-25)
Supplement: Supplementary file 2 [file mbio.01167-25-s0001.docx]

**Table S1: Predicted mutations in independently evolved bacteria populations.** For each mutation, the position, mutation, annotation, affected gene, and gene description are shown. Mutation frequencies are shown as a percentage for each population, with missing entries indicating the mutation was not predicted in that population. Population subscripts indicate the passage of the sample.

**Table S2: Predicted mutations in co-evolved bacteria populations.** For each mutation, the position, mutation, annotation, affected gene, and gene description are shown. Mutation frequencies are shown as a percentage for each population, with missing entries indicating the mutation was not predicted in that population. Population subscripts indicate the passage of the sample.

**Table S3: Predicted mutations in independently evolved phage populations.** For each mutation, the position, mutation, annotation, affected gene, and gene description are shown. Mutation frequencies are shown as a percentage for each population, with missing entries indicating the mutation was not predicted in that population. Population subscripts indicate the passage of the sample.

**Table S4: Predicted mutations in co-evolved phage populations.** For each mutation, the position, mutation, annotation, affected gene, and gene description are shown. Mutation frequencies are shown as a percentage for each population, with missing entries indicating the mutation was not predicted in that population. Population subscripts indicate the passage of the sample.

**Table S5: Predicted mutations in co-evolved bacteria isolates.** For each mutation, the position, mutation, annotation, affected gene, and gene description are shown. Mutation frequencies are shown as a percentage for each isolate, with missing entries indicating the mutation was not predicted in that isolates. Isolates are labelled according to their source population and the number of the isolate e.g., M3-4 represents the 4th isolate from the M3 population.
